# Supplementary material for: Altered microRNA Transcriptome in Cultured Human Liver Cells upon Infection with Ebola Virus
Source: Int J Mol Sci. 2021 Apr 6;22(7):3792. doi: 10.3390/ijms22073792 (PMC8038836; doi:10.3390/ijms22073792)
Supplement: Supplementary file 1 [file ijms-22-03792-s001.zip › Supplementary_File/D_ KEGG_Analysis_Results/16-30nt_pathway_Makona-24h-Huh7_vs_Control-24h-Huh7_up.mature_mirna_targets/hsa_GeneRatioDotPlot.pdf]

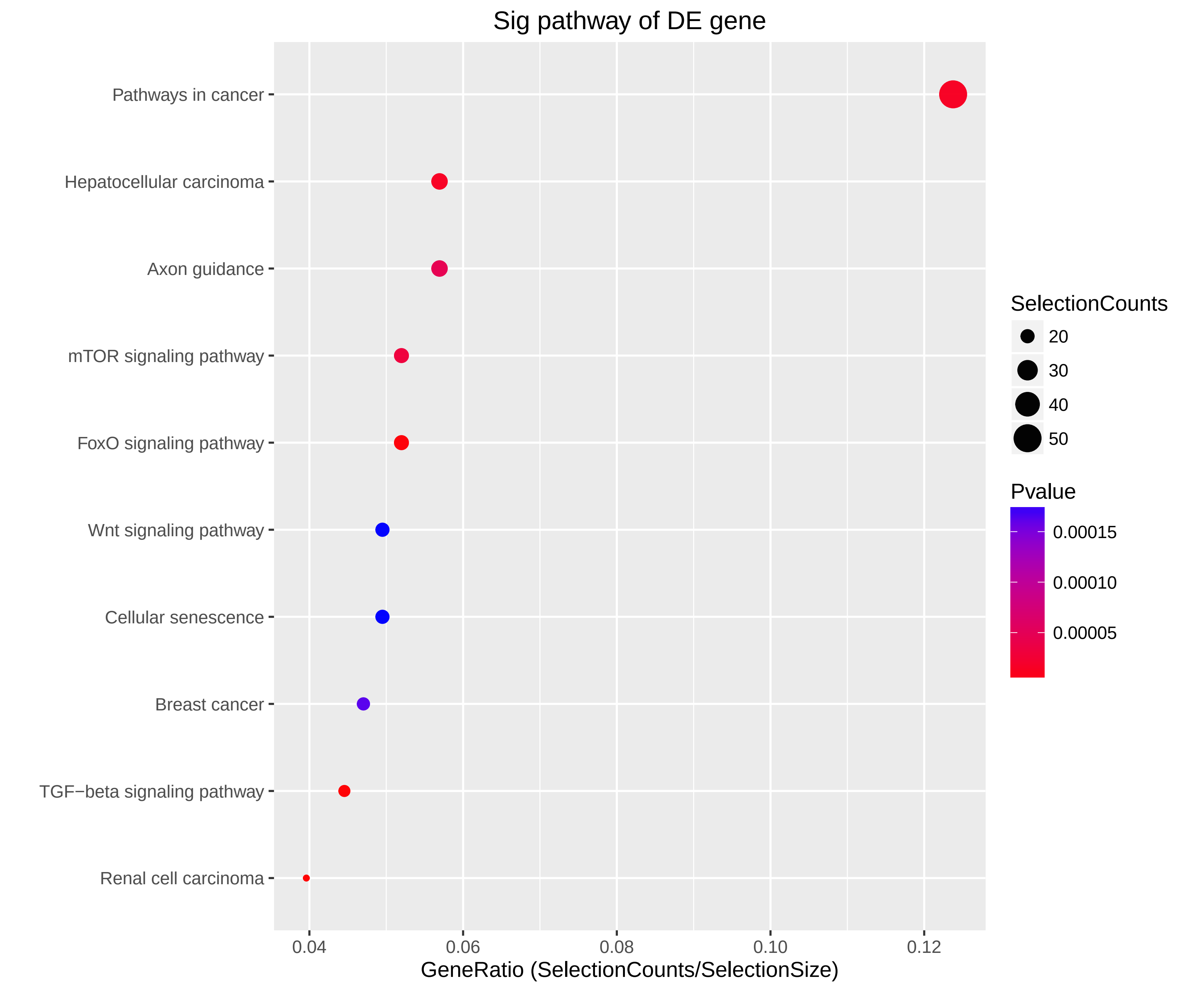

Sig pathway of DE gene
Pathways in cancer
Hepatocellular carcinoma
Axon guidance
mTOR signaling pathway
FoxO signaling pathway
Wnt signaling pathway
Cellular senescence
Breast cancer
TGF−beta signaling pathway
Renal cell carcinoma
0.04
0.06
0.08
0.10
0.12
GeneRatio (SelectionCounts/SelectionSize)
SelectionCounts
20
30
40
50
Pvalue
0.00015
0.00010
0.00005
